# Addition of nonalbumin proteinuria to albuminuria improves prediction of type 2 diabetic nephropathy progression

**DOI:** 10.1186/s13098-017-0267-4

**Published:** 2017-09-06

**Authors:** Jong Ho Kim, Seo Young Oh, Eun Heui Kim, Min Jin Lee, Yun Kyung Jeon, Bo Hyun Kim, Jin Mi Kim, Yong Ki Kim, Sang Soo Kim, In Joo Kim

**Affiliations:** 1 Department of Internal Medicine, Pusan National University Hospital, Busan, South Korea; 2 Biomedical Research Institute, Pusan National University Hospital, Busan, South Korea; 3 Department of Biostatistics, Pusan National University Hospital, Busan, South Korea; 4 Kim Yong Ki Internal Medicine Clinic, Busan, South Korea; 5 Division of Endocrinology and Metabolism, Department of Internal Medicine, Pusan National University Hospital, 179 Gudeok-Ro, Seo-Gu, Busan, 602-739 South Korea

## Abstract

**Background:**

Albuminuria is generally accepted as a sensitive marker of diabetic nephropathy but has limitations in predicting its progression. The aim of this study was to evaluate the use of nonalbumin proteinuria in addition to albuminuria for predicting the progression of type 2 diabetic nephropathy.

**Methods:**

In this retrospective observational study, the urine albumin-to-creatinine ratio (ACR) and the nonalbumin protein-to-creatinine ratio (NAPCR) were measured in 325 patients with type 2 diabetes and estimated glomerular filtration rates (eGFR) ≥30 mL/min/1.73 m^2^. The patients were divided into four groups based on the cutoff points for the urinary ACR (30 mg/g) and NAPCR (120 mg/g). The renal outcomes were chronic kidney disease (CKD) progression and accelerated eGFR decline.

**Results:**

During the 4.3-year follow-up period, 25 (7.7%) patients showed CKD progression and 69 (21.2%) patients showed accelerated eGFR decline. After adjusting for nine clinical parameters, the group with a NAPCR greater than 120 mg/g exhibited higher cumulative incidences of CKD progression (hazard ratio 6.84; *P* = 0.001) and accelerated eGFR decline (hazard ratio 1.95; *P* = 0.011) than the group with a NAPCR < 120 mg/g. In patients with normoalbuminuria, the group with NAPCR levels greater than 120 mg/g also exhibited a higher cumulative incidence than that with NAPCR levels <120 mg/g of CKD progression (hazard ratio 21.82; *P* = 0.005). The addition of NAPCR to ACR improved the model fit for CKD progression and accelerated eGFR decline.

**Conclusion:**

Nonalbumin proteinuria showed additional value over and above that of albuminuria for predicting the progression of CKD in patients with type 2 diabetes.

## Background

The prevalence of diabetes is increasing, and diabetic nephropathy develops in 20–40% of diabetic patients [1, 2]. Type 2 diabetes is the major cause of end-stage renal disease and is associated with high cardiovascular risk and mortality [2, 3]. Early diagnosis of patients at risk for the progression of diabetic nephropathy may reduce the global burden of type 2 diabetes. Albuminuria and the estimated glomerular filtration rate (eGFR) are generally used as established risk markers of renal function [2, 4]. However, albuminuria is detected only after glomerular damage has occurred, and nephropathy is occasionally found before the onset of albuminuria [5, 6]. Therefore, more effective markers are required to delay the progression of diabetic nephropathy.

Proteinuria is composed of albuminuria and nonalbumin proteinuria (NAP). If protein loss to the urine is normal, albumin is considered to be a minor component of the total protein released into urine [7]. It is questionable whether albuminuria alone exhibits useful diagnostic and prognostic power because NAP encompasses various urinary biomarkers and may reflect multiple pathophysiological pathways of renal impairment over the course of diabetic nephropathy [8, 9]. In actuality, NAP may provide a practical picture of the complex pathophysiological status of diabetic nephropathy and could improve the prediction of disease progression [10]. Indeed, we have shown that NAP might be an important marker for the early detection of the progression of diabetic nephropathy, especially in normoalbuminuric patients [11, 12].

Multiple-biomarker approaches based on proteomics are being investigated to overcome the limitations of diagnostic markers for diabetic nephropathy [13, 14]. Unlike plasma, urine can be sampled non-invasively, and proteins in the urine are stable and not subject to rapid degradation [14]. Therefore, urinary proteomics has gained attention as a more accurate diagnostic tool than albuminuria, but it is cost prohibitive. In contrast to urinary proteomics, NAP may be a cost-effective marker for clinical practice.

The role of NAP would be of interest if NAP were to provide additional information on renal outcomes. Therefore, we assessed the value added by NAP when combined with albuminuria to predictions of the progression of chronic kidney disease (CKD) and examined the potential role of the former as an early clinical marker of diabetic nephropathy in patients with type 2 diabetes.

## Methods

### Patients

In this retrospective observational study, 831 outpatients with type 2 diabetes and an estimated glomerular filtration rate (eGFR) ≥ 30 mL/min/1.73 m^2^ were recruited at the Department of Endocrinology and Metabolism, Pusan National University Hospital, Korea, between January 2008 and December 2009. The protocols and consent procedures were approved by the Institutional Review Board of Pusan National University Hospital (approval no. 2013033).

The patients were followed until August 2014. Of the 831 patients, 297 were excluded because they lacked at least 1 year of follow-up data. Among the 534 remaining patients, 139 were excluded because of a history of malignancy (n = 59), cerebrovascular disease (n = 58), cardiovascular disease (n = 5), chronic inflammatory disorder (n = 10), or renal disease other than diabetic nephropathy (n = 7). Seventy more were excluded due to a newly diagnosed neoplasm (n = 32), newly diagnosed cerebrovascular disease (n = 21), acute or chronic disease requiring admission (iatrogenic Cushing’s syndrome, n = 3; hypopituitarism, n = 2; pneumonia, n = 2; hyperglycemic hyperosmolar syndrome, n = 1; hemoptysis, n = 1; cholangitis, n = 1; melena, n = 1; pulmonary tuberculosis, n = 1; nontuberculous mycobacteria, n = 1; toxic hepatitis, n = 1; osteomyelitis, n = 1; pelvic infection, n = 1), or death (n = 1). Ultimately, 325 patients with type 2 diabetes and an eGFR ≥30 mL/min/1.73 m^2^ were included.

### Measurements

GFR was estimated using the equation developed by the Chronic Kidney Disease Epidemiology Collaboration: GFR = 141 × min (serum creatinine/kappa, 1) alpha × max (serum creatinine/kappa, 1) − 1.209 × 0.993 × age × sex × race. For females, the following values were used: sex = 1.018; α = −0.329; κ = 0.7. For males, the following values were used: sex = 1; α = −0.411; κ = 0.9. The eGFR was measured at least twice over at least 12 months of follow up in our clinic. The renal outcomes were CKD progression based on the International Society of Nephrology recommendation statements and accelerated eGFR decline defined as <−3.0 mL/min/1.73 m^2^/year. CKD progression was defined as a decline in GFR category (≥90, stage 1; 60–89, stage 2; 45–59, stage 3a; 30–44, stage 3b; 15–29, stage 4; or <15 mL/min/1.73 m^2^, stage 5), accompanied by a 25% or greater deterioration in the eGFR from the baseline.

Because we obtained random spot urine samples and measured total proteinuria and albuminuria values from each patient at baseline, we were able to estimate the amount of NAP through the following calculation: NAP-to-creatinine ratio (NAPCR) = protein-to-creatinine ratio (PCR) − albumin-to-creatinine ratio (ACR). The lowest detectable level and the coefficient of variation in our laboratory were as follows: for total proteinuria, 0.7 mg/dL and 4.8%, respectively; for albuminuria, 0.2 mg/dL and 7.4%, respectively. Albuminuria was defined using an ACR cutoff of 30 mg/g. There was no reference range or cutoff value for the detection of diabetic nephropathy with NAPCR. Therefore, the cutoff point that provides the best separation of the renal outcomes into two groups was used as an estimate of the unknown cutoff value and the optimal cutoff point for NAPCR was 120 mg/g.

### Statistical analysis

Statistical analyses were performed using SAS software version 9.3 (SAS Institute, Cary, NC, USA) and R version 3.2.2. Data are presented as means ± standard deviation or medians (interquartile range) for skewed variables. Differences between groups were analyzed by one-way analysis of variance (ANOVA), followed by Bonferroni’s test for normally distributed values and by the Kruskal–Wallis test for non-normally distributed values. Chi square testing was used to analyze categorical data as appropriate.

We calculated the optimal cutoff point for NAPCR using a time-to-events approach employing a maximally selected log-rank statistic (with the maxstat package of R 3.2.2). Cumulative incidences of CKD progression and accelerated eGFR decline were evaluated with the Kaplan–Meier method and log-rank test. A Cox regression analysis was performed to assess the effect of several clinical parameters on renal outcomes. Results are presented as hazard ratios (HRs) and 95% confidence intervals (CIs). We used the concordance index (C-index) and Akaike Information Criterion (AIC) as measures of model fit for Cox regression. The model with the higher C-index or the lower AIC is the better fitting model. A two-tailed *P* value <0.05 was considered statistically significant.

## Results

### Baseline patient characteristics

Table 1 summarizes the baseline characteristics of the patients with type 2 diabetes. The patients were categorized into four groups according to the cutoff points of urinary ACR (30 mg/g) and NAPCR (120 mg/g): 146 patients with normoalbuminuria and NAPCR levels below the cutoff point (<120 mg/g), 57 patients with normoalbuminuria and NAPCR levels above the cutoff point (≥120 mg/g), 40 patients with albuminuria and NAPCR < 120 mg/g, and 82 patients with albuminuria and NAPCR ≥ 120 mg/g. The four groups did not significantly differ regarding sex, body mass index (BMI), duration of diabetes, systolic blood pressure (SBP), diastolic blood pressure (DBP), lipid profile, and the frequency of lipid-lowering agents. Glycated hemoglobin (HbA1c) levels were higher in the group with NAPCR ≥ 120 mg/g than in the group with NAPCR < 120 mg/g. Diabetic retinopathy was more frequently observed and more renin–angiotensin system (RAS) inhibitors were administered in the albuminuria group than in the normoalbuminuria group. The levels of eGFR, ACR, and NAPCR significantly differed among the four groups.

**Table 1:** Baseline characteristics of the patients with type 2 diabetes according to urinary ACR and NAPCR cutoff points

	Normoalbuminuria		Albuminuria		P value
	NAPCR < 120 mg/g	NAPCR ≥ 120 mg/g	NAPCR < 120 mg/g	NAPCR ≥ 120 mg/g	
	(n = 146)	(n = 57)	(n = 40)	(n = 82)	
Sex, male (%)	57 (39.0)	17 (29.8)	20 (50.0)	32 (39.0)	0.256
Age, years	54.7 ± 11.4	56.5 ± 9.8	59.7 ± 9.1	54.3 ± 12.0	0.021
BMI, kg/m^2^	25.0 ± 3.6	24.0 ± 2.7	24.3 ± 2.9	24.1 ± 3.9	0.246
Duration of diabetes, years	6.8 ± 5.7	8.1 ± 6.7	9.4 ± 6.1	8.5 ± 7.7	0.059
Hypertension, yes	52 (35.6)	29 (50.9)	22 (55.0)	35 (42.7)	0.071
SBP, mmHg	123 ± 14	123 ± 14	124 ± 15	125 ± 14	0.682
DBP, mmHg	75 ± 10	74 ± 9	73 ± 12	75 ± 10	0.713
HbA1c, %	7.1 ± 1.3	7.6 ± 1.6	7.3 ± 1.4	8.0 ± 1.5	<0.001
Total cholesterol, mg/dL	181 ± 38	184 ± 50	170 ± 44	179 ± 42	0.463
LDL cholesterol, mg/dL	101 ± 31	103 ± 44	91 ± 35	97 ± 33	0.356
HDL cholesterol, mg/dL	48 ± 13	48 ± 13	44 ± 11	48 ± 14	0.252
Triglycerides, mg/dL	137 (89–183)	122 (85–188)	128 (93–197)	150 (106–214)	0.321
Serum creatinine, mg/dL	0.86 ± 0.17	0.80 ± 0.16	0.88 ± 0.25	0.97 ± 0.33	0.001
eGFR, mL/min/1.73 m^2^	87.6 ± 16.7	89.0 ± 14.9	84.0 ± 18.1	79.9 ± 23.1	0.027
ACR, mg/g	9.6 (6.0–14.8)	12.3 (6.7–17.2)	40.7 (37.1–74.7)	131.5 (51.1–499.0)	<0.001
NAPCR, mg/g	75.1 (57.8–91.9)	143.0 (131.4–173.6)	88.0 (65.6–106.0)	203.8 (151.9–295.2)	<0.001
PCR, mg/g	85.7 (67.0–104.9)	153.6 (144.2–190.4)	137.5 (116.5–159.9)	346.9 (213.3–788.4)	<0.001
Diabetic retinopathy, n (%)	18 (19.4)	10 (25.0)	14 (51.9)	34 (55.7)	<0.001
Lipid-lowering agent, n (%)	86 (58.9)	27 (47.4)	23 (57.5)	49 (59.8)	0.452
RAS inhibitor, n (%)	56 (38.4)	25 (43.9)	25 (62.5)	59 (72.0)	<0.001

Data are mean ± standard deviation, medians (interquartile range) for continuous variables and frequencies (percentage) for categorical variables

*BMI* body mass index, *SBP* systolic blood pressure, *DBP* diastolic blood pressure, *HbA1c* glycated hemoglobin, *LDL* low-density lipoprotein, *HDL* high-density lipoprotein, *eGFR* estimated glomerular filtration rate, *ACR* albumin-to-creatinine ratio, *NAPCR* nonalbumin protein-to-creatinine ratio

### Urinary ACR and NAPCR as predictors of CKD progression

Of the 325 patients, 25 (7.7%) showed CKD progression during the follow-up period. The median follow-up period was 4.3 years. In univariate analysis, both albuminuria and NAPCR ≥ 120 mg/g were significantly associated with CKD progression (Table 2). After adjusting for nine clinical parameters, albuminuria (HR 3.43; 95% CI 1.34–8.76; *P* = 0.010) and NAPCR ≥ 120 mg/g (HR 6.84; 95% CI 2.25–20.85; *P* = 0.001) remained significantly associated with CKD progression (Table 2). After additionally adjusting for ACR, NAPCR ≥ 120 mg/g remained significantly associated with CKD progression (HR 5.46; 95% CI 1.64–18.11; *P* = 0.006).

**Table 2:** Univariate and multivariate analysis for CKD progression and accelerated eGFR decline in patients with type 2 diabetes

	Univariate analysis			Multivariate analysis^a^		
	HR	95% CI	P value	HR	95% CI	P value
CKD progression						
Model 1						
ACR < 30	1	Ref.		1	Ref.	
ACR ≥ 30	4.22	1.76–10.11	0.001	3.43	1.34–8.76	0.010
Model 2						
NAPCR < 120	1	Ref.		1	Ref.	
NAPCR ≥ 120	7.59	2.60–22.15	<0.001	6.84	2.25–20.85	0.001
Model 3						
ACR < 30; NAPCR < 120	1	Ref.		1	Ref.	
ACR < 30; NAPCR ≥ 120	18.88	2.27–157.05	0.007	21.82	2.57–185.62	0.005
ACR ≥ 30; NAPCR < 120	11.90	1.23–114.79	0.032	11.62	1.19–113.97	0.035
ACR ≥ 30; NAPCR ≥ 120	27.27	3.60–206.66	0.001	21.40	2.70–169.78	0.004
Accelerated eGFR decline						
Model 1						
ACR < 30	1	Ref.		1	Ref.	
ACR ≥ 30	1.72	1.08–02.76	0.024	1.55	0.92–2.61	0.100
Model 2						
NAPCR < 120	1	Ref.		1	Ref.	
NAPCR ≥ 120	2.01	1.25–3.24	0.004	1.95	1.16–3.26	0.011
Model 3						
ACR < 30; NAPCR < 120	1	Ref.		1	Ref.	
ACR < 30; NAPCR ≥ 120	1.64	0.81–3.31	0.170	1.66	0.81–3.43	0.169
ACR ≥ 30; NAPCR < 120	1.21	0.52–2.84	0.659	1.12	0.47–2.70	0.800
ACR ≥ 30; NAPCR ≥ 120	2.39	1.36–4.17	0.002	2.28	1.21–4.29	0.011

Model 1, vs. normoalbuminuria; model 2, vs. NAPCR levels below 120 mg/g; model 3, vs. normoalbuminuria and NAPCR levels below 120 mg/g

^a^Adjusted for age, sex, duration of diabetes, SBP, LDL, HbA1c, baseline eGFR, RAS inhibitor use and lipid-lowering agent use

**Fig. 1:**
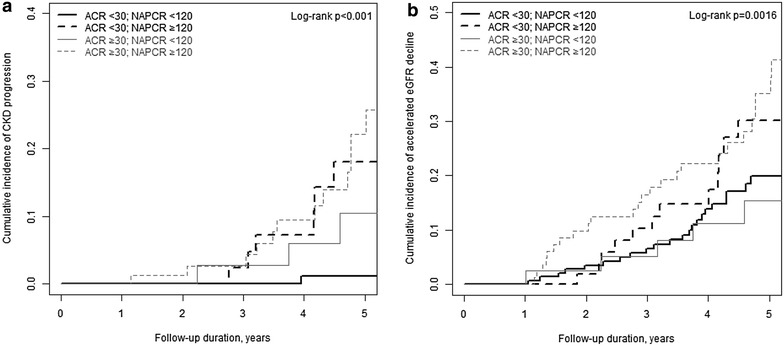
Cumulative incidence of CKD progression (**a**) and accelerated eGFR decline (**b**) using the Kaplan–Meier method and the log-rank test in patients with type 2 diabetes according to urinary ACR and NAPCR cutoff points. *Black lines* ACR below 30 mg/g; *grey lines* ACR above 30 mg/g; *solid lines* NAPCR below 120 mg/g; *dashed lines* NAPCR above 120 mg/g

In model 3, assessing the additional value of NAPCR, a significant difference was observed in the cumulative incidence of CKD progression using the Kaplan–Meier method and log-rank test according to ACR and NAPCR cutoff points (Fig. 1a). After adjusting for nine clinical parameters, the group with NAPCR ≥ 120 mg/g showed a higher cumulative incidence of CKD progression than did the group with NAPCR < 120 mg/g in patients with normoalbuminuria (HR 21.82; 95% CI 2.57–185.62; *P* = 0.005). The group with NAPCR levels greater than 120 mg/g also exhibited a higher cumulative incidence of CKD progression than did that with NAPCR < 120 mg/g in patients with albuminuria (HR 21.40 vs. 11.62; 95% CI 2.70–169.78 vs. 1.19–113.97), but this difference did not reach statistical significance.

We measured C-index and AIC to test the fit of the model for CKD progression (Table 3). The lowest C-index and the highest AIC were observed in the ACR-only model 1. The NAPCR-only model 2 and model 3, which combined ACR and NAPCR, had higher C-indices and lower AIC than the ACR-only model 1. Thus, the model fit for CKD progression was improved by adding NAPCR–ACR.

**Table 3:** Concordance index (C-index) and Akaike Information Criterion (AIC) as measures of model fit for CKD progression and accelerated eGFR decline in patients with type 2 diabetes

	Univariate analysis		Multivariate analysis^a^	
	C-index	AIC	C-index	AIC
CKD progression				
Model 1	0.650	235.363	0.721	243.728
Model 2	0.708	227.673	0.772	235.669
Model 3	0.745	225.506	0.801	234.306
Accelerated eGFR decline				
Model 1	0.552	715.307	0.625	710.887
Model 2	0.585	712.069	0.648	707.140
Model 3	0.592	714.638	0.648	710.365

Model 1, vs. normoalbuminuria; model 2, vs. NAPCR levels below 120 mg/g; model 3, vs. normoalbuminuria and NAPCR levels below 120 mg/g

^a^Adjusted for age, sex, duration of diabetes, SBP, LDL, HbA1c, baseline eGFR, RAS inhibitor use and lipid-lowering agent use

### Urinary ACR and NAPCR as predictors of accelerated eGFR decline

Of the 325 patients, 69 (21.2%) showed accelerated eGFR decline during the follow-up period. The median annual decline in eGFR was −0.1 mL/min/1.73 m^2^/year. In univariate analysis, both albuminuria and NAPCR ≥ 120 mg/g were significantly associated with accelerated eGFR decline (Table 2). After adjusting for nine clinical parameters, only NAPCR ≥ 120 mg/g remained significantly associated with accelerated eGFR decline (HR 1.95; 95% CI 1.16–3.26; *P* = 0.011). After additionally adjusting for ACR, NAPCR ≥ 120 mg/g remained significantly associated with accelerated eGFR decline (HR 1.80; 95% CI 1.04–3.13; *P* = 0.036).

In model 3, assessing the additional value of NAPCR, a significant difference was also observed in the cumulative incidence of accelerated eGFR decline using the Kaplan–Meier method and log-rank test according to ACR and NAPCR cutoff points (Fig. 1b). There was no statistically significant difference after adjusting for nine clinical parameters, but the group with NAPCR ≥ 120 mg/g showed a higher cumulative incidence of accelerated eGFR decline than did that with NAPCR < 120 mg/g in patients with normoalbuminuria (HR 1.66; 95% CI 0.81–3.43; *P* = 0.169). The group with NAPCR levels above 120 mg/g also exhibited a higher cumulative incidence of accelerated eGFR decline than did that with NAPCR < 120 mg/g in patients with albuminuria (HR 2.28 vs. 1.12; 95% CI 1.21–4.29 vs. 0.47–2.70). Additionally, the model fit for accelerated eGFR decline showed that model 1 had the lowest C-index and the highest AIC. Models 2 and 3 showed higher C-indices and lower AIC than model 1. The addition of NAPCR to ACR also improved the model fit for accelerated eGFR decline (Table 3).

## Discussion

In this study, after adjusting for nine clinical parameters, the group with NAPCR levels greater than 120 mg/g exhibited a higher cumulative incidence of CKD progression and accelerated eGFR decline (<−3.0 mL/min/1.73 m^2^/year) than did that with NAPCR < 120 mg/g. In particular, in patients with normoalbuminuria, the group with NAPCR levels greater than 120 mg/g also exhibited a higher cumulative incidence of CKD progression than did that with NAPCR < 120 mg/g. Additionally, the model fit for CKD progression and accelerated eGFR decline was improved by adding NAPCR to ACR.

In Fig. 1, the solid and dashed lines show the difference in the cumulative incidence of CKD progression and accelerated eGFR decline better than the black and gray lines. In other words, the NAPCR cutoff point of 120 may predict the progression of CKD better than the ACR cutoff point of 30. An additional value of NAPCR in predicting the progression of CKD was seen in albuminuric as well as in normoalbuminuric patients (model 3 in Table 2). In patients with normoalbuminuria, the group with NAPCR ≥ 120 mg/g showed a higher cumulative incidence of CKD progression (adjusted HR 21.82; 95% CI 2.57–185.62) and accelerated eGFR decline (adjusted HR 1.66; 95% CI 0.81–3.43) than did that with NAPCR < 120 mg/g. In patients with albuminuria, the group with NAPCR levels greater than 120 mg/g also exhibited a higher cumulative incidence of CKD progression (adjusted HR 21.40 vs. 11.62; 95% CI 2.70–169.78 vs. 1.19–113.97) and accelerated eGFR decline (adjusted HR 2.28 vs. 1.12; 95% CI 1.21–4.29 vs. 0.47–2.70) than did that with NAPCR < 120 mg/g, although this difference did not reach statistical significance. However, as the number of subjects increases, the 95% CI may become narrower, which may lead to significant results. Therefore, future large-scale prospective studies are needed to confirm this finding, and our research in this regard is currently in process.

Albuminuria is commonly used to detect diabetic nephropathy in clinical settings [2, 4]. Urinary albumin is a small component of total urinary protein during normal protein loss, but it becomes the most important protein when protein loss increases [15]. In a study of the general population, the relative ratio of albumin was 21% when the PCR was less than 23 mg/mmol, but it was 73% when the PCR was greater than 89 mg/mmol [16]. As protein loss to the urine decreases, the proportional contribution of albumin to total protein also decreases, and the proportional contribution of nonalbumin protein to total protein may show a relative increase. Therefore, NAP may be used as an effective marker to provide value in addition to that provided by albuminuria to predictions of the progression of diabetic nephropathy.

In general, diabetic patients are constantly exposed to various metabolic and hemodynamic risks [17], and diabetic nephropathy occurs through multiple pathophysiological processes. To reflect the complex pathway that is thought to be related to the progression of diabetic nephropathy, multiple-biomarker approaches using proteomics and metabolomics have been attempted [13, 14, 18]. CKD273 classifier, a panel consisting of 273 urinary peptides, was identified as a good urine proteomics classifier for diagnosing CKD [19], and it was recently validated in an independent cohort as a predictor of the onset of microalbuminuria in normoalbuminuric patients with type 2 diabetes [20]. However, approaches using multiple biomarkers are not cost-effective and are therefore difficult to apply in clinical practice. In contrast, NAP, which includes various urinary proteins associated with renal dysfunction, can be easily measured in urine samples collected at outpatient clinics. Thus, it may be used as a cost-effective marker that can be applied immediately in clinical practice, especially in less developed countries where expensive multiple-biomarker approaches are difficult to use.

Our previous studies identified NAP and six urinary markers, including three tubular markers (KIM-1, NGAL, and L-FABP), two pro-inflammatory markers (IL-18 and YKL-40), and a marker of intrarenal RAS status (angiotensinogen), as biomarkers for the early detection of type 2 diabetic nephropathy [11, 12, 21, 22]. Our recent study suggested that NAP may be a better predictor of the development and progression of CKD than the six urinary markers and ACR in patients with early-stage type 2 diabetic nephropathy [10]. Based on the results of the previous studies, this study was conducted to show the practical value of adding measurement of NAPCR to measurement of ACR alone.

There are several limitations to this study. First, a homogeneous population was used in the present study because our cohort was mono-ethnic and hospital-based. The results of our study need further validation in a multiethnic cohort to evaluate their applicability to broader populations with type 2 diabetes. Second, although urine samples were collected from patients without illness, we measured urinary markers using random spot urine samples at only one time point. Third, diabetic nephropathy was clinically diagnosed without a renal biopsy-proven diagnosis. Fourth, this study was retrospective, and HbA1c levels and RAS inhibitor use differed between groups. We showed the results of adjusting these parameters, but the potential influences of HbA1c levels and RAS inhibitor use on the levels of ACR or NAPCR should be considered. Fifth, it is unclear whether NAP comes from tubular injury alone in patients with diabetic nephropathy characterized by both tubular and glomerular injury. More research is needed to determine whether NAP is a biomarker of tubular injury in clinical conditions with a glomerular protein load, such as diabetic nephropathy.

## Conclusion

Our results suggest that measurement of NAP added to the value of measuring albuminuria alone with regard predicting the progression of CKD in patients with type 2 diabetes; thus, NAPCR could be used with ACR as a biomarker in clinical practice.
